# The potential role of BCL2A1⁺ tissue-resident macrophages in the prognosis of Wilms tumor

**DOI:** 10.1186/s40001-025-02935-3

**Published:** 2025-07-25

**Authors:** Wei Wang, Xianwu Yang, Zhihui Zhu, Zhishen Tang, Yingquan Zhuo, Jun Du, Yuxian Zhu, Xi Luo, Jingjing Xiao, Huajian Gu

**Affiliations:** 1 Department of Pediatric Surgery, Affiliated Hospital of Guizhou Medical University, Guiyang, China; 2 School of Clinical Medicine, Guizhou Medical University, Guiyang, China; 3 Guiyang Maternal and Child Health Hospital, Guiyang, China; 4 Third Department of Hepatobiliary Surgery, Guizhou Provincial People’s Hospital, Guiyang, China

**Keywords:** BCL2A1, Wilms tumor, Tissue-resident macrophages, Prognostic significance, Tumor microenvironment

## Abstract

Tumor-associated macrophages (TAMs) have a significant impact on the prognosis and treatment outcomes of Wilms tumor (WT) patients. To explore the key mechanisms underlying WT progression and immune therapy, this study used CIBERSORT to analyze the immune cell infiltration of 120 WT patients. Combined with single-cell RNA sequencing (scRNA-seq) data, the heterogeneity of macrophages in WT and adjacent tissues was revealed, identifying a subpopulation of tissue-resident macrophages with specific expression of BCL2A1. Further validation through immunohistochemistry (IHC) and immunofluorescence (IF) experiments confirmed the presence of BCL2A1⁺tissue-resident macrophages and elevated BCL2A1 expression is associated with advanced tumors and poor prognosis. Functional enrichment analysis suggests that BCL2A1⁺tissue-resident macrophages may promote WT progression through immune regulation and apoptosis pathways. This study is the first to identify the presence of a BCL2A1⁺tissue-resident macrophage subset in WT and reveal its critical role in tumor progression, potentially providing a novel target for personalized immunotherapy.

## Introduction

Wilms tumor (WT), or nephroblastoma, is the most common malignant renal tumor in children, accounting for over 90% of pediatric renal tumors [1]. The disease primarily affects children around three years old and is characterized by high heterogeneity and aggressiveness [2]. Despite advances in comprehensive treatment strategies combining surgery, radiotherapy, and chemotherapy, which have increased the overall survival rate of WT patients to over 90% [3], approximately 15% of patients still have poor prognosis due to chemotherapy resistance, recurrence, and distant metastasis [4]. Moreover, the existing treatments may lead to long-term adverse effects such as growth retardation, renal dysfunction, and secondary tumors [5]. Therefore, in-depth research into the mechanisms of WT development and progression, as well as the search for new therapeutic targets and strategies, is of significant clinical importance.

The pathogenesis of WT is complex and multifactorial, involving not only genetic mutations and abnormal proliferation of tumor cells but also profound influences from the tumor microenvironment (TME) [6]. The TME is a complex ecosystem composed of tumor cells, immune cells, stromal cells, vasculature, and extracellular matrix components. These elements interact through intercellular signaling, secreted factors, and extracellular vesicles, forming a dynamic network that affects tumor initiation, progression, and metastasis [7]. In the recent years, research on the TME has intensified, with particular focus on its role in tumor progression and therapeutic responses [8]. Immune cells, as critical components of the TME, participate in processes such as tumor cell recognition, killing, and immune evasion [9]. Among them, macrophages occupy a significant position in the TME and are referred to as tumor-associated macrophages (TAMs). Their quantity and functional state significantly influence tumor growth, angiogenesis, immune suppression, and metastasis [10]. In WT, while considerable research has focused on the molecular biological characteristics of tumor cells, studies on immune cells in its microenvironment, especially macrophages, remain relatively limited [11]. Existing studies suggest that WT development may be related to changes in the immune microenvironment, but the specific mechanisms are not yet clear [12]. Therefore, in-depth exploration of the characteristics of macrophages in WT and their roles in tumor progression is crucial for understanding the pathogenesis of WT and identifying new therapeutic targets.

Macrophages exhibit a spectrum of functional phenotypes, ranging from M1 (pro-inflammatory) to M2 (anti-inflammatory) states. The recent studies have shown that macrophage polarization exists as a continuum, with various intermediate and hybrid phenotypes that play different roles in inflammation, tissue repair, and tumor progression [13, 14]. M1 macrophages secrete pro-inflammatory cytokines, such as IL-12 and TNF-α, exerting anti-tumor and anti-infection effects [15]. In contrast, M2 macrophages secrete anti-inflammatory cytokines like IL-10 and TGF-β, promoting tissue repair, angiogenesis, and tumor growth [16]. In the TME, M2 macrophages usually predominate, facilitating tumor immune evasion and progression [17]. BCL2A1 (B-cell lymphoma 2-related protein A1) is an anti-apoptotic protein belonging to the Bcl-2 family [18]. It is primarily expressed in myeloid cells and activated lymphocytes, participating in cell survival and anti-apoptotic regulation [19]. In addition, BCL2A1 is highly expressed in various tumors and is associated with drug resistance and poor prognosis. In melanoma, Haq et al. found that overexpression of BCL2A1 leads to resistance to BRAF inhibitors [20]. In acute myeloid leukemia, BCL2A1 may become a novel therapeutic target for treating FLT3-ITD/D835 mutant AML [21]. In glioblastoma, BCL2A1 promotes tumor progression and affects patient prognosis by participating in TAM infiltration [22]. However, the presence and distribution characteristics of BCL2A1 and macrophages in WT, and their impact on the TME, have not been studied.

In the recent years, the rapid development of single-cell RNA sequencing (scRNA-seq) technology has provided a powerful tool for revealing the heterogeneity of immune cells in the TME [23]. By conducting high-throughput sequencing of gene expression in individual cells within tumor tissues, different cell subpopulations can be precisely identified, and their functional characteristics, developmental trajectories, and interactions can be understood [24]. In various tumors, scRNA-seq has been successfully applied to discover new immune cell subpopulations and reveal their roles in tumor development and progression [25].

This study investigates the heterogeneity of macrophages in the WT microenvironment by analyzing high-throughput sequencing and scRNA-seq data [26]. We identify different macrophage subpopulations, focusing on the identification and biological functions of the BCL2A1⁺tissue-resident macrophage subpopulation. We aim to understand the expression of BCL2A1 in these subpopulations and explore its relationship with tumor progression and the immune microenvironment. Through experiments, such as IHC and IF, we validate BCL2A1⁺macrophages to clarify their presence and distribution characteristics in WT. In addition, combined with survival analysis, we evaluate the impact of BCL2A1 expression levels on the prognosis of WT patients. We hypothesize that BCL2A1⁺macrophages play a crucial role in the progression of WT by modulating the immune microenvironment and facilitating tumor growth. By identifying and characterizing these macrophages, we aim to explore their potential as novel therapeutic targets for personalized immunotherapy. This study provides new theoretical evidence for developing individualized treatment strategies targeting macrophages in WT [27].

## Materials and methods

### Clinical specimen collection

Between 2015 and 2023, we obtained 48 unstained, paraffin-embedded WT tissue Sects. (4 μm thick) from the Department of Pathology at the Affiliated Hospital of Guizhou Medical University. The specimens comprised 8 cases of stage I, 20 cases of stage II, 13 cases of stage III, and 7 cases of stage IV. All cases were pathologically confirmed as WT by certified pathologists. Informed consent was obtained from all patients and their guardians. The study protocol was approved by the Ethics Committee of the Affiliated Hospital of Guizhou Medical University (Approval No: 2024-249).

### Immune infiltration analysis

High-throughput sequencing data (FPKM format) of WT were downloaded from the TARGET database (https://ocg.cancer.gov/programs/target). After preprocessing and excluding samples lacking complete clinical information, 120 tumor samples with comprehensive clinical data were retained. Immune cell infiltration was analyzed using the CIBERSORT package in R (v4.2.3). CIBERSORT estimates the relative proportions of 22 immune cell types based on the LM22 gene signature matrix using a support vector regression algorithm [28]. The results were visualized using the ggpubr and ggplot2 packages.

### Single-cell RNA sequencing data analysis

Public scRNA-seq data of WT were obtained from the Human Cell Atlas (HCA) database (https://www.humancellatlas.org/) [29]. The data processing was performed using the Seurat package (v5.0.0) in R (v4.2.3). After constructing a Seurat object, cells were filtered based on the following criteria: nFeature_RNA > 200, nFeature_RNA < 7500, percent.mt < 5%, and percent.HB < 3%. Batch effects were corrected using the Harmony algorithm. The data were normalized using ScaleData, dimensionality reduction was performed using principal component analysis (PCA), and clustering analysis was conducted using the UMAP method. Differential genes of each cell subpopulation were identified using the FindAllMarkers function, and subpopulations were annotated based on specific marker genes.

### Immunohistochemistry and immunofluorescence

#### Immunohistochemistry

Paraffin-embedded tissue sections were deparaffinized in xylene (G2150, Solarbio, Beijing, China) and rehydrated through graded alcohols. Antigen retrieval was performed under high pressure for 10 min using EDTA antigen retrieval solution (ZLI-9066, ZSbio, Beijing, China). Endogenous peroxidase activity was blocked in a 37 °C water bath for 20 min using peroxidase blocking reagent (PV-9000, ZSbio, Beijing, China), followed by blocking with goat serum (SAP-9100, ZSbio, Beijing, China) for 30 min at room temperature. Primary antibodies (see supplementary table) were added and incubated overnight at 4 °C. The following day, an amplification reagent and HRP-conjugated goat antirabbit/mouse IgG polymer (PV-9000, ZSbio, Beijing, China) were applied and incubated in a 37 °C water bath for 20 min. DAB substrate (ZLI-9018, ZSbio, Beijing, China) was used for color development for 1 min, and the nuclei were counterstained with hematoxylin (G1080, Solarbio, Beijing, China) for 2 min. After dehydration, sections were mounted with neutral resin (G8590, Solarbio, Beijing, China). Slides were observed and photographed using an Olympus (VS200) laser confocal microscope or a Nikon (A1) microscope with default parameters, auto exposure, and magnification recorded in the resulting images.

#### Immunofluorescence

Paraffin-embedded tissue sections were deparaffinized in xylene and rehydrated through graded alcohols, followed by antigen retrieval under high pressure for 10 min using EDTA antigen retrieval solution. Sections were blocked with goat serum (SAP-9100, ZSbio, Beijing, China) for 30 min, and primary antibodies (see supplementary table) were added and incubated overnight at 4 °C in the dark. The following day, fluorescently labeled secondary antibodies were applied and incubated in the dark in a 37 °C water bath for 20 min. Nuclei were stained with DAPI (C0065, Solarbio, Beijing, China) for 10 min, and sections were mounted with anti-fade mounting medium (S2100, Solarbio, Beijing, China). Slides were observed and photographed using an Olympus (VS200) laser confocal microscope or a Nikon (A1) microscope with default parameters, auto exposure, and magnification recorded in the resulting images.

#### Quantitative analysis of immunohistochemical staining intensity

After immunohistochemical staining, five random high-power fields (200 × magnification) were selected from each tumor stage group for image acquisition, avoiding necrotic areas and technical artifacts. FILI software was used to quantitatively analyze the staining intensity of BCL2A1. IHC images were imported into the software and color separation was performed to highlight BCL2A1-positive staining areas (brown). The separated images were converted to grayscale for quantitative analysis. Threshold settings were uniformly adjusted to distinguish positive staining areas from the background. The measurement tool was used to calculate the average grayscale value of the positive area, representing the staining intensity. Staining intensity data from different stages (I–IV) were compiled, and *t* tests were used to compare differences between stages, with a significance level set at *P* < 0.05.

### Spatial transcriptomics analysis

Spatial transcriptomics sequencing data of mouse kidney tissue were obtained from the GEO database (https://www.ncbi.nlm.nih.gov/geo/) with accession numbers GSM6009058, GSM6009059, and GSM6009060. The SRA Toolkit (v2.11.0) was used to download and convert SRA files to FASTQ format. Data preprocessing and analysis were performed using Space Ranger software (v3.0.0) from 10 × Genomics. The mouse reference genome sequence (Mus_musculus.GRCm39.dna.primary_assembly.fa.gz) and gene annotation file (Mus_musculus.GRCm39.110.gtf.gz) were downloaded from the Ensembl database to construct a reference dataset using the mkref function in Space Ranger. Each sample was aligned and quantified using the Space Ranger count command with default parameters. The generated spatial expression matrices were used for subsequent integrated analysis.

In R (v4.0.5), the Seurat package (v3.2.3) was used to integrate the spatial transcriptomics data with the scRNA-seq data. Following an anchor-based workflow, the FindTransferAnchors function was used to generate anchor sets, and the TransferData function was utilized to transfer cell type information from the single-cell data to the spatial data. The SpatialFeaturePlot function was employed to visualize the spatial expression patterns of specific genes (e.g., Bcl2a1a).

### Functional enrichment analysis

Gene Ontology (GO) [30]and Kyoto Encyclopedia of Genes and Genomes (KEGG) [31] pathway enrichment analyses were performed on the differentially expressed genes using the clusterProfiler package in R (v4.2.3). GO analysis included biological processes (BP), cellular components (CC), and molecular functions (MF). Enrichment was assessed using the hypergeometric test, and multiple testing correction was performed using the Benjamini–Hochberg method, with a significance threshold of *p* < 0.05.

KEGG pathway enrichment analysis was conducted similarly using the clusterProfiler package. Pathways with adjusted *p* values less than 0.05 were considered significant.

### Pseudotime analysis

To explore cellular developmental trajectories, pseudotime analysis was performed using the monocle package on the single-cell data processed by Seurat (v5.0.0) [32, 33]. Genes expressed in fewer than three cells were excluded. Library size normalization was conducted using the estimateSizeFactors function, and gene dispersion was estimated with the estimateDispersions function. Genes with mean expression greater than 0.1 and variance exceeding the empirical dispersion were selected. Dimensionality reduction was performed using the DDRTree method, and cells were ordered along the pseudotime trajectory using the orderCells function. Developmental trajectory plots were generated.

### Cell interaction analysis

CellChat (v1.6.1) was used in R (v4.2.3) for cell interaction analysis. ScRNA-seq data were quality-controlled, normalized, feature-selected, and clustered using the Seurat package (v5.0.0) [34]. Cell subpopulations were annotated based on known marker gene expression patterns. The CellChatDB database was used to construct cell interaction networks. The compareInteractions function compared signaling pathway activities between different cell subpopulations, and significant pathways were ranked using the rankNet function. The computeCommunProbPathway function calculated interaction probabilities for each pathway. Bubble plots were generated using the netVisual_bubble function to display the main cell interaction relationships.

### High-dimensional weighted gene co-expression network analysis (hdWGCNA)

We performed weighted gene co-expression network analysis (hdWGCNA) on the merged scRNA-seq data using the hdWGCNA package (version 0.3.03) [35, 36]. First, we utilized the MetacellsByGroups function to construct meta cells for each sample and each cell cluster, respectively. Subsequently, we sequentially executed the functions TestSoftPowers, ConstructNetwork, ModuleEigengenes, ModuleConnectivity, and RunModuleUMAP for network construction, module identification, and dimensionality reduction analyses.

### Survival analysis

#### Survival analysis based on BCL2A1 expression

WT patients were stratified into high and low BCL2A1 expression groups based on the median expression value. Kaplan–Meier survival analysis was performed using the survival package in R, and the log-rank test was used to evaluate survival differences between the two groups, with a significance level set at *p* < 0.05. Survival curves were plotted using the survminer package.

#### Time-dependent ROC curve analysis

To assess the predictive ability of BCL2A1 as a prognostic biomarker, time-dependent ROC curve analysis was conducted using the timeROC package on patients'overall survival time, survival status, and BCL2A1 expression levels. AUC values at 6, 8, and 12 months were calculated, and curves were visualized using the ggplot2 package. An AUC value closer to 1 indicates stronger predictive ability.

#### Univariate Cox regression analysis

Clinical information of patients, including overall survival time, survival status, gender, age at diagnosis, tumor stage, and BCL2A1 expression level, was extracted. Univariate Cox proportional hazards regression analysis was performed using the coxph function in the survival package to evaluate the impact of each variable on prognosis. The results reported hazard ratios (HR), 95% confidence intervals, and *p* values, with a significance level set at *p* < 0.1.

## Results

### Immune infiltration analysis reveals the prominent role of macrophages in the WT microenvironment

**Fig. 1:**
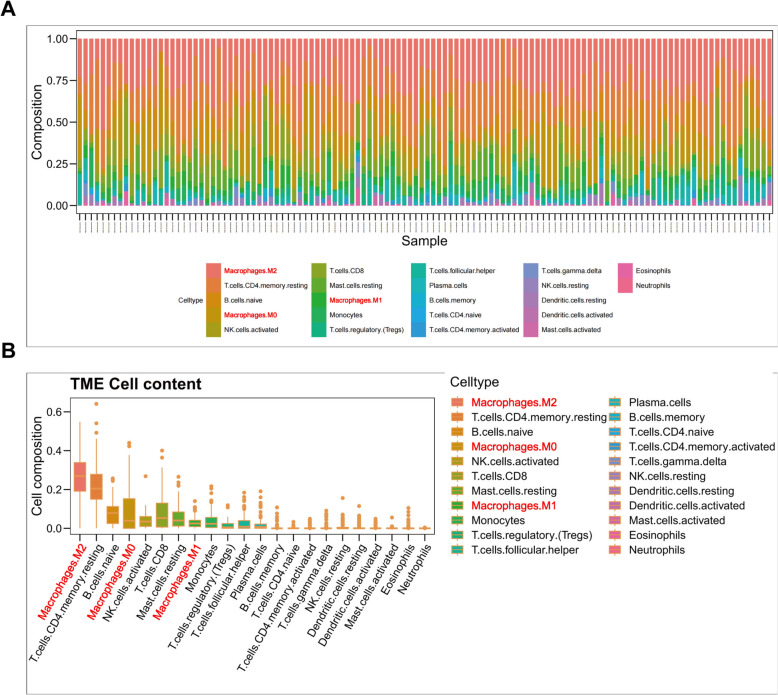
Immune infiltration analysis revealing the prominent role of macrophages in the WT microenvironment. **A**, Cell abundance plot showing the proportional distribution of different immune cells within the WT microenvironment. **B**, Box plot illustrating the relative proportions of various immune cells in WT samples

To investigate the distribution of immune cells within the TME of WT, we utilized the CIBERSORT algorithm for immune cell analysis. High-throughput sequencing data from 120 WT patients were obtained from the TARGET database and processed through CIBERSORT. The results showed a substantial presence of M2 macrophages in WT samples (Fig. 1A), with their relative proportion significantly higher than that of other immune cells such as T cells, NK cells, and B cells (Fig. 1B).

### Single-cell data analysis reveals the complexity of macrophage infiltration in WT

**Fig. 2:**
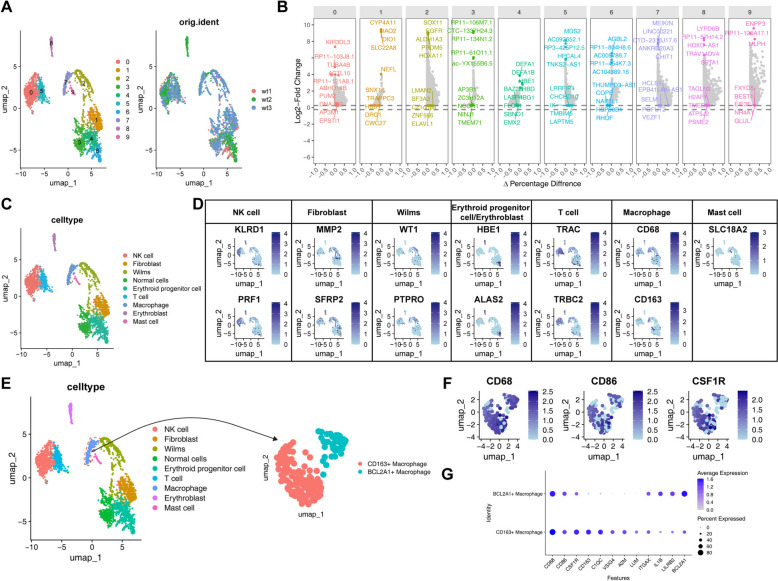
Single-cell data analysis reveals the complexity of macrophage infiltration in WT. **A**, UMAP plot showing identified cell subpopulations in WT samples. **B**, The grouped dot plot displays the top 10 differential genes for each cell subtype. **C**, UMAP annotation plot showing cell subpopulation annotations based on differential genes. **D**, UMAP plot illustrating characteristic genes and distribution of annotated cell subpopulations. **E**, Reclustering of macrophages in WT tissue. **F**, UMAP plots showing the expression of CD68, CD86, and CSF1R in macrophage subpopulations. **G**, Bubble plot displaying the expression levels of macrophage-related genes in CD163⁺and BCL2A1⁺macrophages

To delve deeper into the heterogeneity of macrophages in WT, we acquired scRNA-seq data from WT patients in the HCA database. After quality control, 2274 high-quality cells were obtained. Unsupervised dimensionality reduction techniques, including PCA and UMAP [37], were applied to cluster all cells and identify distinct cellular subpopulations in WT (Fig. 2A). Initially, 10 distinct populations were identified, and through differential gene expression analysis and identification of specific gene markers, we performed gene annotation (Fig. 2B, C). During manual annotation, we merged certain populations based on the marker gene expression. Specifically, populations 4, 6, and 8 expressed the marker genes HBE1 and ALAS2 simultaneously, so these populations were grouped together due to their shared marker expression. Furthermore, population 3 did not show specific marker gene expression and, after referring to the annotation information from the sequencing data article, was classified as normal cells. As a result, we identified 7 distinct subpopulations, which include NK cells (KLRD1, PRF1), fibroblasts (MMP2, SFRP2), Wilms tumor cells (WT1, PTPRO), erythroid progenitor cells/erythroblasts (HBE1, ALAS2), T cells (TRAC, TRBC2), macrophages (CD68, CD163), and mast cells (SLC18A2) (Fig. 2D). Quality control results for each cell subpopulation validated the reliability of the annotations (Figure S2A).

Further clustering analysis of the annotated macrophage subpopulation revealed two distinct macrophage subclusters in WT (Fig. 2E). Both subclusters highly expressed typical macrophage markers CD68, CD86, and CSF1R [38], confirming their macrophage identity (Fig. 2F). First, we identified M2 TAMs specifically expressing the CD163 gene (Fig. 2G). This subpopulation was highly enriched in WT, consistent with the results of the CIBERSORT immune infiltration analysis. In addition, we identified a novel macrophage subpopulation that, while expressing CD68, CD86, and CSF1R, specifically exhibited high expression of the BCL2A1 gene (Fig. 2G). The discovery of this subpopulation provides a new direction for research.

In summary, this study reveals for the first time two critical macrophage subpopulations in WT: the known pro-tumor M2 macrophages and the newly identified BCL2A1⁺macrophage subpopulation.

### Functional and differentiation differences between intratumoral BCL2A1⁺and CD163⁺macrophages

To validate the presence of BCL2A1⁺macrophages within WT, we performed IHC and IF analyses on patient paraffin-embedded sections, using CD68 and CD163 as macrophage markers [39].

**Fig. 3:**
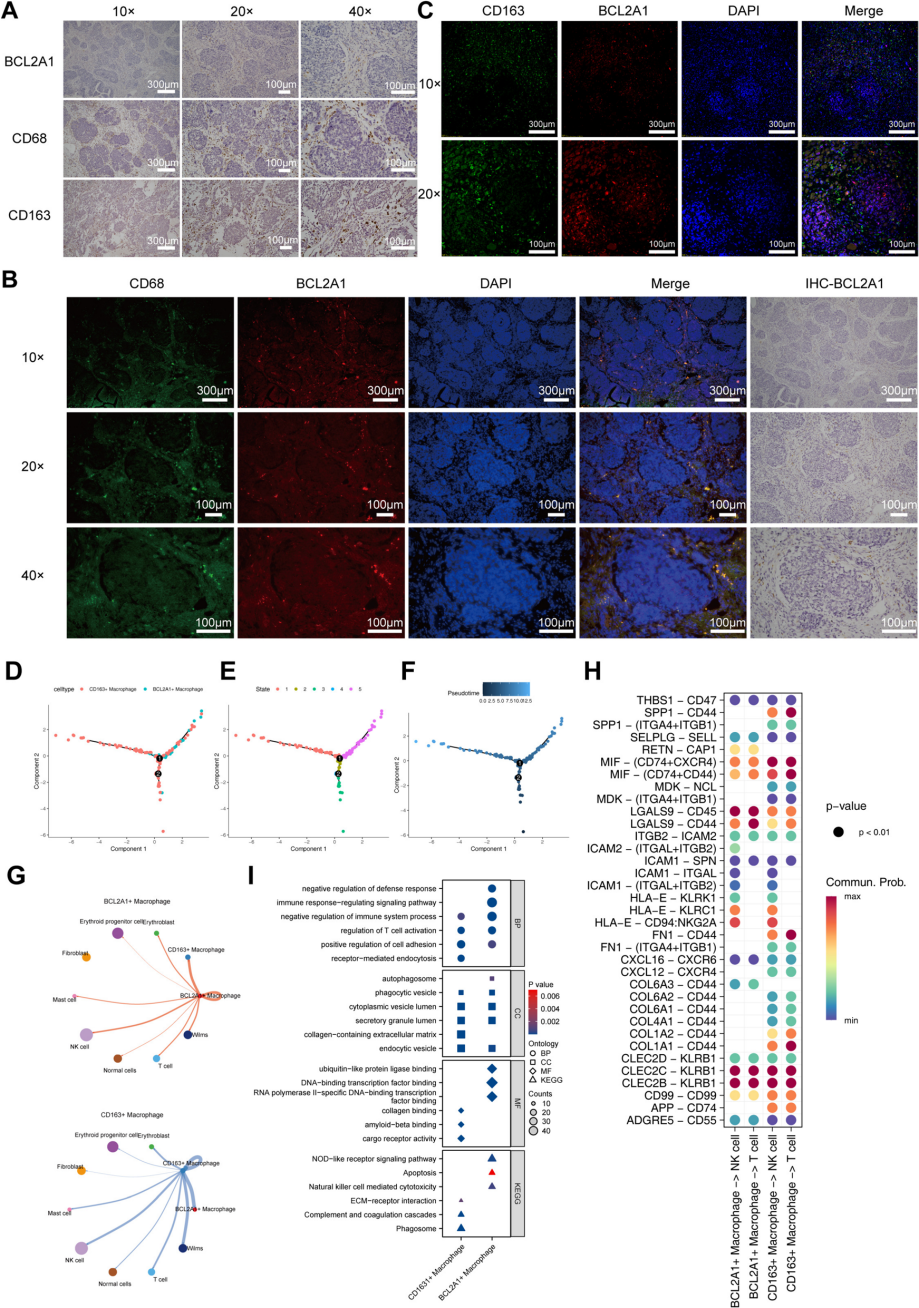
Functional and differentiation differences between intratumoral BCL2A1⁺and CD163⁺macrophages. **A**, IHC staining results of BCL2A1, CD68, and CD163 in WT samples. **B**, IF co-staining showing co-localization of BCL2A1 and CD68 in tumor cells. **C**, IF co-staining showing co-localization of BCL2A1 and CD163. **D**, Pseudotime analysis showing distribution differences between BCL2A1⁺and CD163⁺macrophages. **E**–**F**, Pseudotime trajectories of BCL2A1⁺and CD163⁺macrophages. **G**, Cell communication diagrams between BCL2A1⁺macrophages, CD163⁺macrophages, and other cells. **H**, Bubble plot showing key receptor–ligand pairs between BCL2A1⁺macrophages, CD163⁺macrophages, T cells, and NK cells. **I**, Grouped bar chart displaying GO and KEGG enrichment analysis results of BCL2A1⁺and CD163⁺macrophages

IHC results demonstrated significant expression of BCL2A1, CD68, and CD163 within the tumors, with BCL2A1 exhibiting a distribution pattern highly similar to that of CD68 and CD163, primarily localized in the tumor stroma region (Fig. 3A). This spatial concordance suggests that BCL2A1⁺cells align with macrophage localization. Further confirming the existence of BCL2A1⁺macrophages, IF co-staining showed substantial co-localization of BCL2A1 and CD68 at the same sites (Fig. 3B), supporting our identification of the BCL2A1⁺macrophage subset. In contrast, co-staining of BCL2A1 and CD163 did not overlap and displayed different spatial distributions (Fig. 3C). This finding is consistent with scRNA-seq data, indicating that BCL2A1⁺macrophages and CD163⁺M2 macrophages differ in function and distribution, representing distinct functional subsets.

To explore the developmental trajectories and cell–cell communication of these two macrophage types within the TME, we conducted pseudotime analysis and cell communication analysis. The results revealed that BCL2A1⁺macrophages and CD163⁺macrophages occupy different positions along the dimensionality reduction axes (Fig. 3D). BCL2A1⁺macrophages were mainly concentrated in State 5 (Fig. 3E), suggesting they may be in a fully differentiated or specific functional state. As pseudotime progressed, CD163⁺macrophages transitioned from early to late states (Fig. 3F), reflecting dynamic changes in their functions. In addition, cell communication analysis indicated that interactions between BCL2A1⁺macrophages and natural killer (NK) cells were prominent (Fig. 3G). In contrast, CD163⁺macrophages engaged in complex communication networks with multiple cell types. Further analysis of key receptor–ligand pairs showed that CD163⁺macrophages participated in various intricate interactions involving multiple signaling pathways. Notably, the LGALS9-CD45 receptor–ligand pair between BCL2A1⁺macrophages and NK cells exhibited significant prominence (Fig. 3H).

To further elucidate the functional characteristics of the two types of macrophages, we performed GO BP, CC, MF, and KEGG pathway enrichment analysis. BCL2A1⁺macrophages were significantly enriched in the"immune response-regulating signaling pathway"(GO:0002764),"negative regulation of defense response"(GO:0031348),"autophagosome"(GO:0005776),"DNA-binding transcription factor binding"(GO:0140297), as well as the"apoptosis"(hsa04210) and"natural killer cell mediated cytotoxicity"(hsa04650) pathways (Fig. 3I). This is consistent with the known apoptotic regulatory function of BCL2A1. In contrast, CD163⁺macrophages were enriched in the"receptor-mediated endocytosis"(GO:0006898),"collagen-containing extracellular matrix"(GO:0062023),"amyloid-beta binding"(GO:0001540), and ECM–receptor interaction (hsa04512) pathways, suggesting that they may play an important role in antigen processing.

In summary, BCL2A1⁺and CD163⁺macrophages exhibit distinct functional characteristics and developmental trajectories in WT. BCL2A1⁺macrophages may influence tumor progression through apoptosis regulation and modulation of immune responses, whereas CD163⁺macrophages may be involved in antigen presentation. These findings further validate the reliable presence of these two macrophage types within tumors.

### Origin and tissue-resident distribution characteristics of BCL2A1⁺macrophages in normal kidney tissue

To investigate the origin of the newly identified BCL2A1⁺macrophages and their distribution characteristics in normal renal tissue, we analyzed scRNA-seq data from adjacent normal tissues of WT patients, combined with IHC and multiplex IF techniques for validation. In addition, we utilized spatial transcriptomics data of mouse kidney tissue-resident macrophages to further clarify the tissue-resident properties and spatial distribution patterns of Bcl2a1a⁺macrophages.

**Fig. 4:**
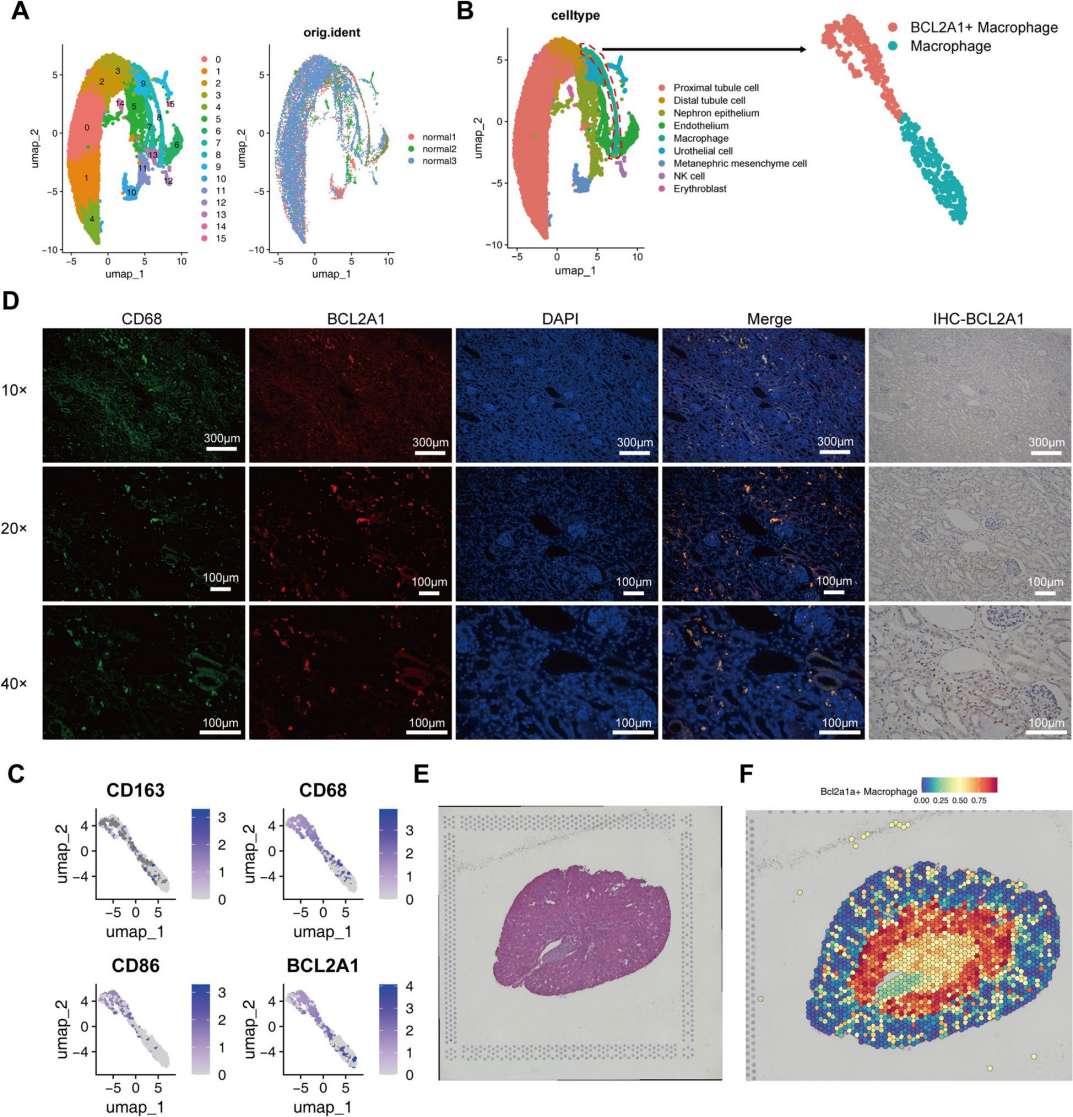
Origin and tissue residency of BCL2A1⁺macrophages in normal kidney tissue. **A**, UMAP plot showing cell subpopulations in normal adjacent tissues of WT. **B**, Reclustering of macrophages in adjacent normal tissues. **C**, UMAP plot showing the expression of CD163, CD68, CD86, and BCL2A1 in macrophage subpopulations. **D**, IF and IHC results showing co-localized expression of BCL2A1 and CD68 in adjacent normal tissues, and IHC showing BCL2A1 expression in renal tubular epithelial cells. **E**, HE-stained image of spatial transcriptomics slice of mouse kidney. **F**, Spatial distribution map of Bcl2a1a-expressing macrophages combining scRNA-seq data and spatial transcriptomics chips

Single-cell sequencing analysis identified multiple cell subpopulations (Fig. 4A), including erythroid progenitor cells (HBE1, ALAS2), NK cells (NKG7, KLRD1), mesonephric mesenchymal cells (PAX2, SLC23A3), urothelial cells (UPK1A, UPK2), macrophages (CD68, CD163), endothelial cells (PTPRB, PECAM1), and nephron epithelial cells (CLCNKB, SLC12A1) (Figure S2B). Based on marker genes, each subpopulation was annotated (Fig. 4B), and heatmaps of the top 50 marker genes for each subpopulation were generated (Figure S2C). After extracting the macrophage subpopulation for further clustering analysis, results showed that in adjacent normal tissues, BCL2A1⁺macrophages were the predominant macrophage subpopulation (Fig. 4B), and no subpopulations with significant expression of M2 macrophage–associated genes were found (Fig. 4C).

IHC results demonstrated that BCL2A1 was expressed in the renal tubular epithelial cells of adjacent normal tissues, with punctate BCL2A1-positive regions similar to those observed in tumor tissues (Fig. 4D), indicating that BCL2A1 is also expressed in macrophages of normal tissue. Dual IF staining confirmed that BCL2A1 co-localized with the macrophage marker CD68 within the same cells in adjacent normal tissue (Fig. 4D), confirming that BCL2A1⁺cells are macrophages and preliminarily suggesting that these cells are tissue-resident macrophages in normal renal tissue.

Furthermore, spatial transcriptomic analysis of mouse kidney tissue [40] supported this conclusion. By integrating mouse tissue-resident macrophages expressing the Bcl2a1a gene (Figure S2D–F) with spatial transcriptomic tissue sequencing chips (Fig. 4E), we found that tissue-resident macrophages expressing Bcl2a1a were mainly distributed in the renal medulla region near the renal pelvis (Fig. 4F) (Figure S1A–B), displaying clear tissue-resident characteristics.

In summary, we confirmed the presence and distribution of BCL2A1⁺macrophages in normal renal tissue and preliminarily revealed their identity as tissue-resident macrophages.

### Commonalities and differences of BCL2A1⁺macrophages from different tissue origins

**Fig. 5:**
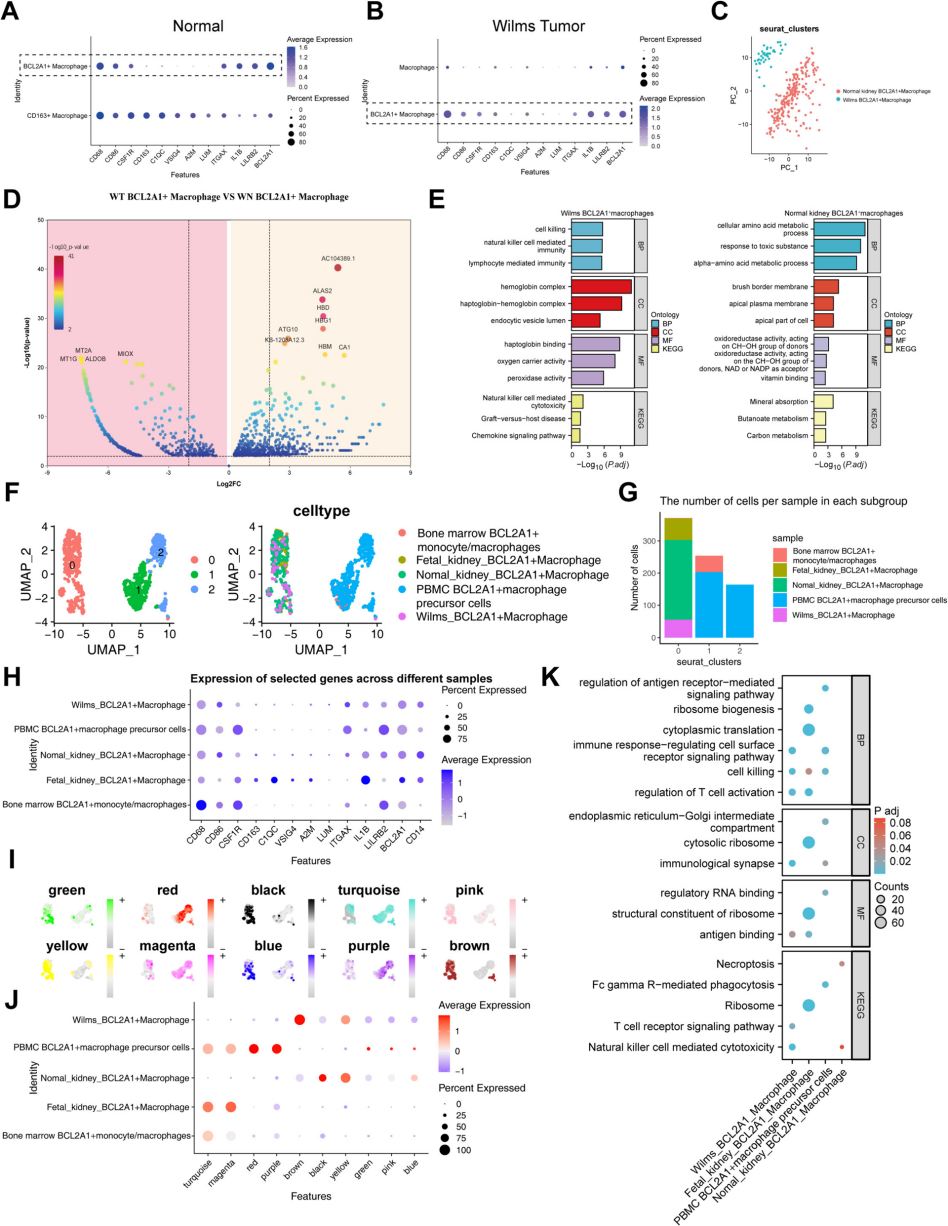
Commonalities and differences of BCL2A1⁺macrophages from different tissues. **A**-**B**, Bubble plots showing the expression of macrophage marker genes in BCL2A1⁺macrophages from tumor and adjacent normal tissues. **C**, PCA showing the distribution of BCL2A1⁺macrophages from tumor and normal tissues in principal component space. **D**, Volcano plot showing differential genes of BCL2A1⁺macrophages between tumor and normal tissues. **E**, Bar chart displaying KEGG and GO enrichment analysis results of the differential genes. **F**, UMAP plot displaying the dimensionality reduction clustering of distinct macrophage clusters. **G**, Bar chart showing the number of cells distributed across different macrophage clusters. **H**, Bubble plot illustrating the expression of signature genes in various macrophage clusters. **I**, UMAP plot indicating the expression distribution of module central genes across different macrophage clusters. **J**, Module activity estimation in different macrophage clusters using the hdWGCNA algorithm. **K**, Bubble plots showing GO and KEGG enrichment results for each macrophage cluster

To compare the gene expression characteristics and biological functions of BCL2A1⁺macrophages in WT and adjacent normal tissues, we conducted principal component analysis (PCA), differential gene expression analysis, and KEGG and GO functional enrichment analyses. scRNA-seq data showed that BCL2A1⁺macrophages in tumor and normal tissues had highly similar expression of macrophage marker genes (such as CD68 and CD163) (Fig. 5A–B), indicating consistent fundamental macrophage characteristics. However, PCA revealed that these two cell types were distinctly separated in the principal component space (Fig. 5C). Differential gene expression analysis (Fig. 5D) uncovered gene expression differences between BCL2A1⁺macrophages from the two tissue origins. KEGG and GO enrichment analyses showed that BCL2A1⁺macrophages in tumors were enriched for genes related to immune functions, particularly involving NK cells, and lymphocyte-mediated immune response pathways (Fig. 5E), consistent with previous results. In contrast, BCL2A1⁺macrophages in normal tissues were enriched for genes related to metabolic functions, mainly involving amino acid and small molecule metabolic processes.

To further explore the tissue-resident characteristics of BCL2A1⁺macrophages, we analyzed scRNA-seq data from fetal kidney (Figure S1C), pediatric bone marrow (Figure S1D), and peripheral blood (Figure S1E). The results showed that these tissues contained BCL2A1-positive cells (Figure S1F–H). Based on the relevant markers and tissue origins, the cells were described as Wilms BCL2A1⁺macrophages, Fetal kidney BCL2A1⁺macrophages, normal kidney BCL2A1⁺macrophages, PBMC BCL2A1⁺macrophage precursor cells, and bone marrow BCL2A1⁺monocyte/macrophages. After integrating BCL2A1⁺cell data from WT, adjacent normal tissue, fetal kidney, pediatric bone marrow, and peripheral blood, UMAP dimensionality reduction clustering revealed that BCL2A1⁺macrophages from WT, adjacent normal tissue, and fetal kidney clustered together, while BCL2A1⁺monocyte/macrophages from bone marrow and BCL2A1⁺macrophage precursor cells from peripheral blood formed an independent group (Fig. 5F–G). This finding suggests that BCL2A1⁺acrophages in WT and normal kidney tissues are highly similar at the molecular level to those in fetal kidney, supporting their potential as tissue-resident macrophages.

Further analysis revealed that BCL2A1⁺cells from all five tissue origins expressed both CD68 and BCL2A1 (Fig. 5H). BCL2A1⁺macrophages in WT, adjacent normal tissue, and fetal kidney also expressed CD14, while BCL2A1⁺cells in bone marrow and peripheral blood did not express CD14. Notably, in bone marrow and peripheral blood, there were subpopulations expressing CD68 and CD14 but not BCL2A1, or expressing only CD68 without CD14 (Figure S1G–H). In contrast, in fetal kidney, only cell populations co-expressing CD68 and BCL2A1 were present (Figure S1F).

To further explore the functional significance of these differences, we performed single-cell weighted gene co-expression network analysis on the merged BCL2A1⁺cells, identifying ten co-expression modules (Figure S2G). BCL2A1⁺macrophages from WT were predominantly enriched in the brown module (Fig. 5I–J). GO and KEGG enrichment analyses indicated that genes in this module were primarily involved in immune-related processes, including T cell receptor signaling and natural killer cell-mediated cytotoxicity (Fig. 5K), consistent with the previous findings. In contrast, BCL2A1⁺macrophage precursor cells in peripheral blood were mainly enriched in the purple and red modules (Fig. 5I–J). Enrichment analyses suggested that genes in these modules were related to the regulation of antigen receptor-mediated signaling pathways, immune response regulation, and Fc gamma receptor-mediated phagocytosis (Fig. 5K). BCL2A1⁺macrophages in fetal kidney were enriched in the turquoise and magenta modules (Fig. 5I–J). Enrichment analysis revealed that these genes were mainly involved in ribosome biogenesis, cytoplasmic translation, and ribosomal structural components (Fig. 5K). KEGG pathway analysis further indicated that these genes were highly enriched in ribosome-related pathways.

In summary, BCL2A1⁺macrophages from different tissue origins exhibit differences in molecular characteristics and functions. BCL2A1⁺macrophages in WT and fetal kidney share similar tissue-resident characteristics.

### Expression of BCL2A1 in WT and its prognostic implications

To investigate the impact of BCL2A1 gene expression levels on the prognosis of WT and to evaluate its feasibility as a potential prognostic factor, we analyzed the expression levels of BCL2A1 in WT and normal tissues. Combining survival analysis, time-dependent receiver operating characteristic (ROC) analysis [41], and univariate regression analysis, we explored the correlation between BCL2A1 and patient outcomes.

**Fig. 6:**
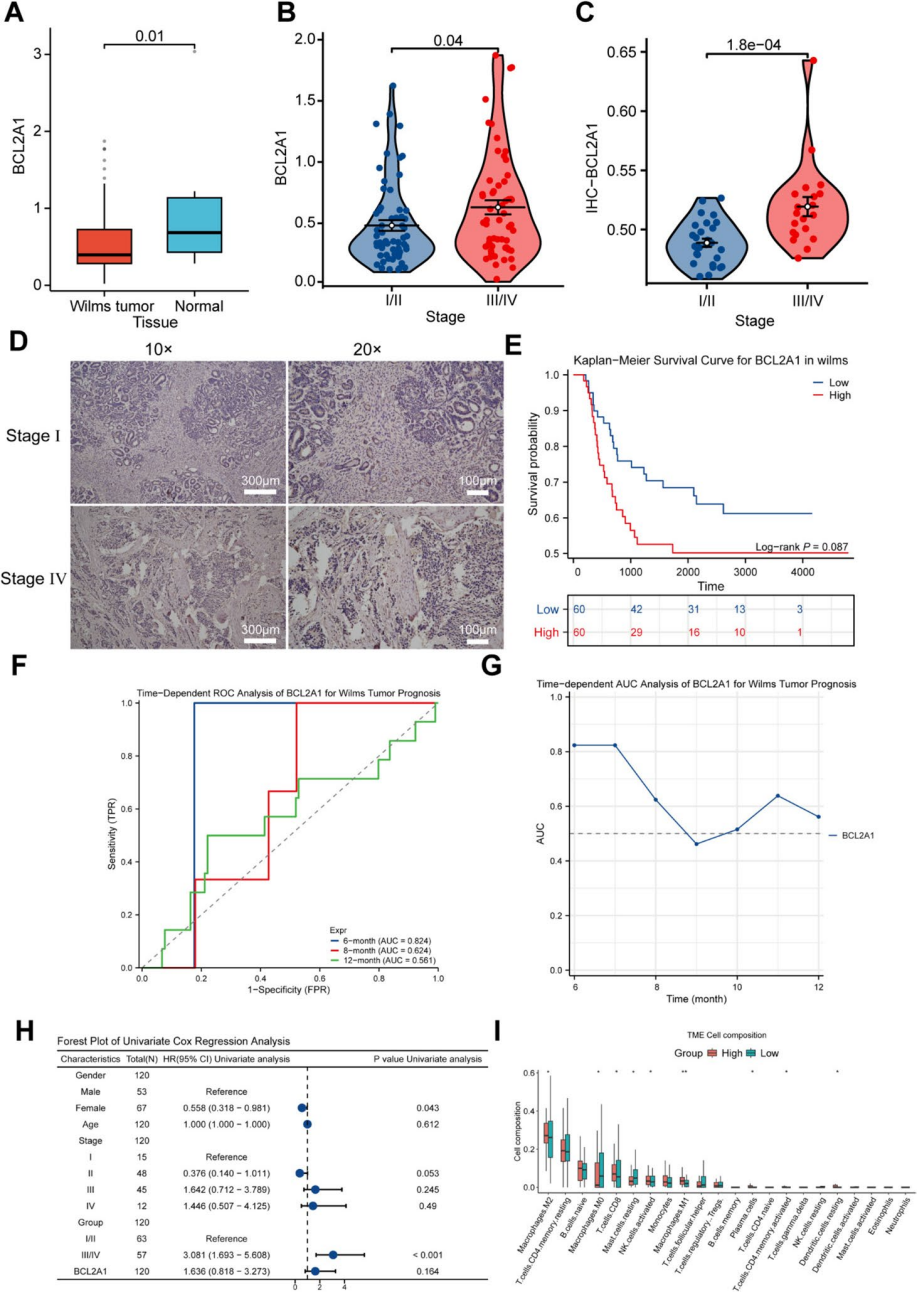
Expression of BCL2A1 in WT and its prognostic impact. **A**, Box plot showing that BCL2A1 expression in WT is lower than in normal tissue (*P* < 0.05). **B**, Violin plot showing that BCL2A1 expression levels increase with tumor stage (*P* < 0.05). **C**, **D**, Immunohistochemical staining and violin plot showing that the positive staining rate of BCL2A1 in stage III and IV patients is significantly higher than in stage I and II patients (*P* < 0.05). **E**, Kaplan–Meier survival curves comparing survival rates between high and low BCL2A1 expression groups (*P* > 0.05). **F**–**G**, Time-dependent ROC analysis plots of BCL2A1. **H**, Results of univariate Cox regression analysis. **I**, Grouped box plots showing the relationship between BCL2A1 expression and immune cell components in the TME

Initially, the results showed that BCL2A1 expression was significantly lower in WT tissues compared to normal tissues (Fig. 6A), suggesting that BCL2A1 does not function as an oncogene in WT. However, significant differences in BCL2A1 expression were observed across different tumor stages. Patients with stage III and IV tumors exhibited significantly higher BCL2A1 expression levels than those with stage I and II tumors, with statistically significant differences (Fig. 6B). Immunohistochemical staining of paraffin-embedded sections from 48 WT patients revealed that the positive staining rate of BCL2A1 progressively increased with advancing tumor stages. The positive rate in stage I and II patients was markedly lower than that in stage III and IV patients (*p* < 0.001), further confirming the correlation between BCL2A1 expression and tumor progression (Fig. 6C,D).

To assess the relationship between BCL2A1 expression levels and WT patient prognosis, we performed Kaplan–Meier survival analysis. The results indicated that the survival rate in the high BCL2A1 expression group was lower than that in the low expression group, although the difference did not reach statistical significance (log-rank *P* = 0.087, Fig. 6E). However, the separation trend of the survival curves suggested that high BCL2A1 expression might be associated with poorer prognosis.

Subsequently, we conducted a time-dependent ROC analysis of BCL2A1. The results showed that the area under the curve at 6 months was 0.824, indicating the best prognostic performance. However, as time progressed, the AUC gradually decreased to 0.624 at 8 months and 0.561 at 12 months (Fig. 6F–G), suggesting that BCL2A1 has higher predictive efficacy in the early stages, but its effectiveness diminishes over time.

In addition, to further verify whether BCL2A1 is an independent prognostic factor, we performed univariate Cox regression analysis. The results demonstrated that tumor stage (stage III and IV) could serve as an independent prognostic factor with significant statistical differences (HR = 3.081, 95% CI 1.693–5.608, *P* < 0.001, Fig. 6H). However, BCL2A1 expression levels did not exhibit a significant independent prognostic effect in univariate analysis (*P* = 0.164).

Finally, we analyzed the relationship between different immune cell components in the TME and BCL2A1 expression. The results showed that high BCL2A1 expression was significantly associated with an increased proportion of M2 macrophages, accompanied by specific changes in immune cells, such as T cells and NK cells (*P* < 0.05) (Fig. 6I).

In summary, BCL2A1 expression in WT is related to tumor stage and has certain prognostic predictive value in the short term. However, its role as an independent prognostic factor has not been fully established. Further studies are needed to validate the potential of BCL2A1 as a prognostic indicator.

## Discussion

In this study, we integrated immune infiltration analysis with scRNA-seq, for the first time, the presence of BCL2A1⁺tissue-resident macrophages in WT. Our results demonstrate that BCL2A1⁺macrophages are present in both tumor tissues and adjacent normal tissues and are associated with poor prognosis in WT patients, suggesting their potential role in tumor progression. IHC and IF further confirmed the existence and spatial distribution of BCL2A1⁺macrophages in WT and normal tissues. Notably, BCL2A1⁺macrophages exhibited significant differences from CD163⁺macrophages within tumor tissues. CD163⁺macrophages engage in complex cellular communication networks with various cell types, potentially influencing the tumor immune microenvironment by modulating multiple signaling pathways involved in antigen processing and T cell activation. In contrast, BCL2A1⁺macrophages predominantly interact with natural killer (NK) cells, particularly through the LGALS9–CD45 receptor–ligand pair. This specific intercellular communication may play a critical role in regulating NK cell function and, consequently, the immune response to the tumor. These distinctions indicate that BCL2A1⁺and CD163⁺macrophages possess different functional characteristics in WT.

Comparative analysis of BCL2A1⁺macrophages from tumor and adjacent normal tissues revealed highly similar expression of macrophage marker genes (e.g., CD68, CD163). However, differential gene expression and functional enrichment analyses showed that tumor-associated BCL2A1⁺macrophages are enriched in pathways related to immune response regulation, apoptosis, and NK cell–mediated cytotoxicity, suggesting their involvement in immune modulation within the TME. Integrative analysis of BCL2A1⁺macrophages from WT, adjacent normal tissues, fetal kidney, bone marrow, and peripheral blood indicated that those from tumors and fetal kidney share high molecular similarity but differ markedly from those in peripheral blood and bone marrow. This finding supports the notion that BCL2A1⁺macrophages in tumors exhibit tissue-resident properties and may play unique immunoregulatory roles within the TME. The influence of different tissue environments on the function of BCL2A1⁺macrophages underscores the importance of microenvironmental factors in determining macrophage function, aligning with the descriptions by Ginhoux and Guilliams regarding the functional diversity of tissue-resident macrophages [42]. These insights provide a new perspective on the role of BCL2A1⁺macrophages in tumor progression and lay the groundwork for developing individualized immunotherapeutic strategies targeting macrophages.

Interestingly, we found that the overall expression level of BCL2A1 in WT tissues is significantly lower than in normal tissues, suggesting that BCL2A1 does not act as an oncogene in the traditional sense within WT [19, 20]. However, the high expression of BCL2A1 in specific macrophage subpopulations significantly impacts tumor progression. Elevated levels of BCL2A1 were observed in patients with advanced stages (III and IV), and a trend toward decreased survival rates was noted in patients with high BCL2A1 expression. Although survival analysis did not reach statistical significance, time-dependent ROC analysis indicated that BCL2A1 has high efficacy in short-term prognostic prediction. These findings suggest that BCL2A1⁺macrophages may promote WT progression by influencing the TME. This observation challenges the traditional understanding of the role of BCL-2 family proteins in tumor development and highlights the complexity of tumor biology. It emphasizes the need to consider not only the expression of tumor-related genes in cancer cells but also their expression patterns in various cell types within the TME. The discovery of BCL2A1⁺tissue-resident macrophages expands our understanding of tumor-associated macrophage (TAM) heterogeneity. Traditional classifications have focused on M1 and M2 macrophages, where M1 macrophages are considered pro-inflammatory and anti-tumorigenic, and M2 macrophages are viewed as anti-inflammatory and pro-tumorigenic [43]. However, this simplified classification does not fully explain the diverse roles of TAMs in tumor progression. The differential expression patterns of BCL2A1⁺macrophages in tumors and normal tissues further support the"subversion hypothesis,"which posits that the TME can"subvert"tissue-resident macrophages to acquire abilities that promote tumor growth and metastasis [44]. Our findings highlight the necessity for in-depth studies on macrophage heterogeneity, echoing the work of Zhang et al., who identified a special role for tissue-resident macrophages in hepatocellular carcinoma [45].

Despite these important findings, our study has limitations. The relatively small sample size may affect the statistical significance of our analyses. In addition, while we validated the presence of BCL2A1⁺macrophages and their association with prognosis through bioinformatic analyses and histological validation, we lack in-depth exploration of their functional mechanisms. Future research should focus on elucidating these mechanisms and developing targeted therapies for this macrophage subpopulation. Inhibiting the expression or function of BCL2A1 may reduce the pro-tumor effects of these macrophages, thereby improving patient outcomes. For instance, venetoclax, the first approved BCL-2 inhibitor, has achieved significant progress in treating hematological malignancies [46]. Its successful application in chronic lymphocytic leukemia and acute myeloid leukemia has offered new hope to patients [47, 48]. With ongoing research, the indications for venetoclax are expected to expand to additional tumor types. By optimizing combination immunotherapies to overcome resistance, venetoclax's role in cancer treatment may become more prominent [49]. In subsequent studies, we will explore the application of venetoclax in WT immunotherapy using in vitro and in vivo models, aiming to aid patients with poor prognosis due to chemotherapy resistance, recurrence, and distant metastasis [4]. This approach aligns with emerging tumor immunotherapy strategies that seek to achieve anti-tumor effects by modulating immune cells within the TME [50].

In conclusion, our study is the first to reveal the presence and potential functions of BCL2A1⁺tissue-resident macrophages in WT, enriching the understanding of macrophage heterogeneity within the TME. Targeting BCL2A1⁺tissue-resident macrophages offers new insights into the mechanisms of WT development and progression and provides potential targets for developing individualized venetoclax-based macrophage-directed immunotherapies in the future.

## Data Availability

The datasets presented in this study are available in online repositories. Single-cell sequencing data for Wilms tumor can be accessed in the HCA database (https://data.humancellatlas.org/) under the project titled Single-cell transcriptomes from human kidneys reveal the cellular identity of renal tumors. Single-cell sequencing data for fetal kidneys, pediatric peripheral blood, and pediatric bone marrow are available in the GEO database (https://www.ncbi.nlm.nih.gov/geo/) under the following accession numbers: fetal kidneys (GSE114530), pediatric peripheral blood (GSM6250006, GSM6250007, GSM6250008), and pediatric bone marrow (GSM4664009, GSM4664010, GSM4664011, GSM4664012).
